# Bi-potential hPSC-derived Müllerian duct-like cells for full-thickness and functional endometrium regeneration

**DOI:** 10.1038/s41536-022-00263-2

**Published:** 2022-11-23

**Authors:** Lin Gong, Nanfang Nie, Xilin Shen, Jingwei Zhang, Yu Li, Yixiao Liu, Jiaqi Xu, Wei Jiang, Yanshan Liu, Hua Liu, Bingbing Wu, XiaoHui Zou

**Affiliations:** 1 Department of Gynecology, the First Affiliated Hospital, Zhejiang University School of Medicine, Hangzhou, P.R. China; 2 Dr. Li Dak Sum & Yip Yio Chin Center for Stem Cell and Regenerative Medicine, School of Medicine, Zhejiang University, Hangzhou, P.R. China; 3 Central Laboratory, the First Affiliated Hospital, Zhejiang University School of Medicine, Hangzhou, P.R. China; 4 Key Laboratory of Tissue Engineering and Regenerative Medicine of Zhejiang Province, School of Medicine, Zhejiang University, Hangzhou, P.R. China; 5 Chu Kochen Honors College, Zhejiang University, Hangzhou, P.R. China; 6 International Institutes of Medicine, the Fourth Affiliated Hospital, Zhejiang University School of Medicine, Yiwu, Zhejiang China

**Keywords:** Stem-cell differentiation, Stem-cell research

## Abstract

Stem cell-based tissue regeneration strategies are promising treatments for severe endometrial injuries. However, there are few appropriate seed cells for regenerating a full-thickness endometrium, which mainly consists of epithelia and stroma. Müllerian ducts in female embryonic development develop into endometrial epithelia and stroma. Hence, we first generated human pluripotent stem cells (hPSC)-derived Müllerian duct-like cells (MDLCs) using a defined and effective protocol. The MDLCs are bi-potent, can gradually differentiate into endometrial epithelial and stromal cells, and reconstitute full-thickness endometrium in vitro and in vivo. Furthermore, MDLCs showed the in situ repair capabilities of reconstructing endometrial structure and recovering pregnancy function in full-thickness endometrial injury rats, and their differentiation fate was revealed by single-cell RNA sequencing (scRNA-seq). Our study provides a strategy for hPSC differentiation into endometrial lineages and an alternative seed cell for injured endometrial regeneration.

## Introduction

The endometrium is part of the female reproductive system and plays a vital role in maintaining regular menstruation, allowing embryo implantation, and housing a developing baby^1^. It mainly consists of endometrial epithelial and stromal cells, which synthesise and secrete substances necessary for normal physiological functions^2^. Surgery or infection can cause endometrial injuries and obstruct the regeneration capability of endometrial epithelia and stroma, hindering the repair process and leading to fibrosis and scar formation, which can result in concerns related to menstrual disorders, intrauterine adhesions (IUA), infertility, and abortion^3–5^. Although conventional methods such as surgery and hormonal treatments are available to overcome the aforementioned issues, the repair of severe or widespread endometrial injuries remains challenging^6^. Therefore, researchers have attempted to find the optimal method to promote endometrial repair after injury to restore the native structure and function of the endometrium.

Stem cell-based therapy is a novel approach for the treatment of severe endometrial injury^7^. Multiple stem cell types have been studied for their role in endometrial repair. For example, adipose-derived mesenchymal stem cell sheets and bone marrow-derived mesenchymal stem cell (BMSC)-loaded scaffolds have been shown to improve the repair of severely damaged uterus endometrium in rat models^8–10^. Human umbilical cord-derived mesenchymal stem cells injected via the rat tail vein^11^ or loaded on the autocross-linked hyaluronic acid gel in in situ research on rhesus monkeys^12^ both repaired the endometrial injury. However, these mesenchymal stem cells (MSCs) have limited differentiation potential, differentiating into stromal-like cells at most^13^. Furthermore, Ong et al. demonstrated that BMSCs do not contribute to endometrial cell lineages (epithelial, stromal or endothelial cells) in their chimeric mouse models and suggested that other studies may have misidentified immune cells^14^.

Human pluripotent stem cells (hPSCs), namely human embryonic stem cells (hESCs) and human induced pluripotent stem cells (hiPSCs), are capable of unlimited self-renewal and multipotent differentiation and provide unprecedented opportunities for cell therapies against intractable diseases and injuries^15^. hPSC-derived cells have been receiving increasing attention and have been found to exhibit repair effects in endometrial injury. hESC-derived endometrium-like cells from a co-culture system combined with collagen scaffolds retrieved the structure and function of uterine horns in rats^16^. hiPSC-derived MSCs (hiMSC)-loaded scaffolds repaired the damaged endometrium and restored its ability to support embryos^17^. Nonetheless, a simple differentiation strategy to induce hPSCs into an endometrium-specific lineage has not been defined, and no seed cells can replenish both endometrial epithelial cells and stromal cells for full-thickness endometrial repair.

The endometrium of the female reproductive duct develops from the Müllerian duct epithelium of the embryo, which can differentiate into endometrial epithelia and stroma^18^. Müllerian ducts (also called paramesonephric ducts) are derived from the intermediate mesoderm (IM), the coelomic epithelium invagination, which forms an early duct structure and undergoes mesenchymal-epithelial transformation^19,20^. Several studies have developed defined protocols with developmental signals to induce hPSCs into the IM stage, which provides references for early-stage differentiation^21–23^. Simulating the Müllerian duct development pathway to induce further differentiation of hPSC-derived IM cells would result in Müllerian duct-like cells (MDLCs) in vitro. The Wnt/β-catenin signalling pathway plays a key regulatory role in the formation and differentiation of Müllerian ducts, as demonstrated by *Wnt4*, *Wnt5a*, *Wnt7a* and *Wnt9b* in animal models^18,19,24–26^. Therefore, hPSC-derived IM cells might be further induced and differentiated by activating Wnt signalling, a crucial signal for Müllerian duct development.

Hence, in this study, a simple, defined protocol was established to induce stepwise differentiation of hPSCs into the endometrial primordium, which we named “Müllerian duct-like cells”. MDLCs show the ability of endometrial-specific lineage differentiation and the potency of bidirectional differentiation, which can generate endometrial organoids/tissues with an epithelium–stroma structure in in vitro 3D culture and in vivo. Furthermore, they reconstructed the endometrial structure and recovered pregnancy function in rat models of full-thickness endometrial injury. Moreover, scRNA-seq analysis demonstrated the fate of MDLCs in vivo during the repair of endometrial injury. This study established a workflow to generate hPSC-derived MDLCs and demonstrated their potential as effective seed cells for the clinical treatment of uterine diseases, providing a feasible treatment strategy for the repair of endometrial injury.

## Results

### Stepwise differentiation of hPSCs into MDLCs

**Fig. 1:**
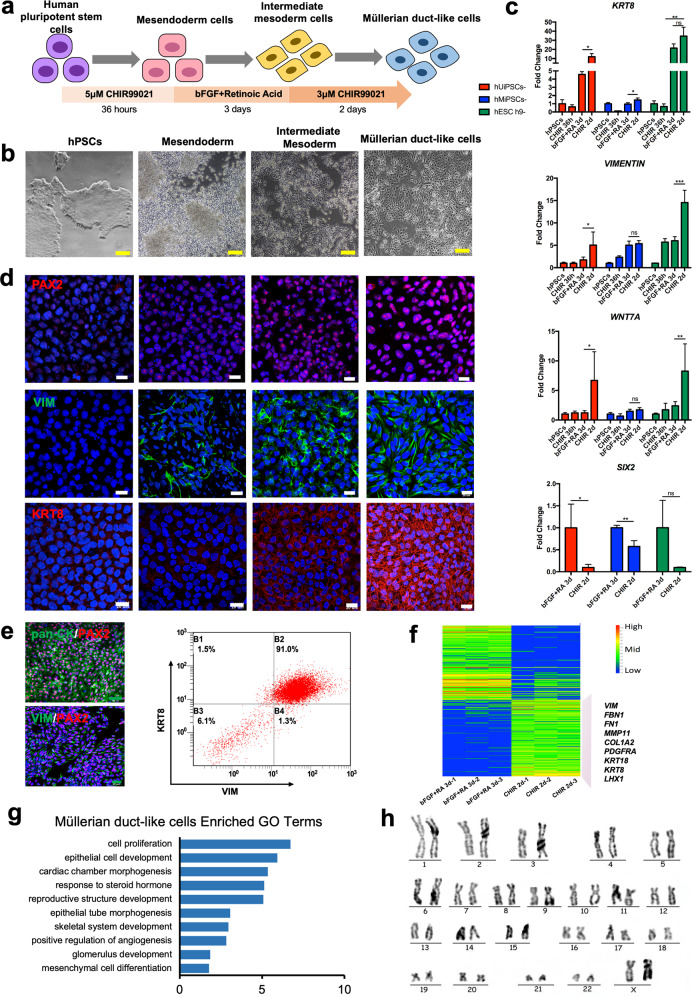
Differentiation and characterisation of hPSC-derived Müllerian duct-like cells. **a** Diagram of stepwise differentiation of hPSCs into MDLCs with chemicals. MDLCs Müllerian duct-like cells. **b** Light microscopic observation of morphology in each stage. Yellow scale bars, 500 μm. **c** Expression level of *KRT8*, *VIM*, *WNT7A* and *SIX2* in three cell lines at each stage. hUiPSCs human urine-derived induced pluripotent stem cells, hMiPSCs human monocyte-derived induced pluripotent stem cells. (*VIM*, *VIMENTIN*; *KRT8*, *KERATIN 8*) (data represent mean ± SEM, *N* = 3. *P < 0.05; **P < 0.01; ***P < 0.001; ns (no significance), *P* > 0.05 by one-way ANOVA and unpaired, two-tailed Student’s *t* test). **d** Immunofluorescence examination of multiple markers, PAX2, VIM and KRT8 in each stage. White scale bars, 20 μm. DAPI (blue). **e** Verification of epithelial/mesenchymal hybrid features of CHIR 2d-treated cells by immunofluorescence and flow cytometry. (pan-CK, pan-Cytokeratin) Green scale bars, 40 μm. **f** RNA-seq analysis of IM cells and CHIR 2d-treated cells, gene expression profiling by heatmap; representative upregulated genes are listed in this figure. (*P* value < 0.01). **g** Biological process of CHIR 2d-treated cells shown by GO. **h** Karyotype of hPSC-derived MDLCs.

To generate hPSC-derived IM cells, we first applied the induction method described by Lam et al.^22^. In brief, hPSCs (two hiPSC cell lines, one hESC h9 cell line) (*OCT4, NANOG*) were treated with 5 μM CHIR99021 for 36 h (CHIR 36 h) to efficiently differentiate into mesendodermal cells (*BRACHYURY, MIXL1*). They then differentiated into IM cells (*PAX2, OSR1, LHX1*) with the induction of 100 ng/ml basic fibroblast growth factor (bFGF) and 10 nM retinoic acid (RA) for 3 days (bFGF+RA 3d) (Fig. 1a and Supplementary Fig. 1a). Bulk RNA-seq was performed to analyse transcriptomic changes during cell differentiation. Principal component analysis (PCA) indicated that the cells in the three stages were distinct (Supplementary Fig. 1b). DEseq2 analysis showed that the pluripotent marker gene *SOX2* was significantly downregulated and that the mesoderm genes *BRACHYURY* (also known as *T*), *LHX1*, *TBX6* and *BMP4* were significantly upregulated after CHIR 36-h treatment (Supplementary Fig. 1c). Gene ontology (GO) analysis identified enrichment of somitogenesis, heart looping, paraxial mesoderm formation, primitive steak formation, and mesendoderm development (Supplementary Fig. 1d). After bFGF+RA 3d treatment, *BRACHYURY* and *TBX6* were downregulated, but the marker genes of intermediate mesoderm *PAX2*, *PAX8*, *LHX1* and *KDR* were significantly upregulated. The epithelial cell markers *KRT8* and *KRT18* and mesenchymal markers *VIMENTIN (VIM)* and *fibronectin 1* (*FN1*) were also highly expressed (Supplementary Fig. 1e). GO analysis identified the enrichment of embryonic skeletal system formation, positive regulation of heart contraction, ureteral bud formation, embryonic placental development, and mesenchymal-to-epithelial transition involved in metanephros morphogenesis (Supplementary Fig. 1f). These results demonstrate that IM cells were successfully generated from hPSCs.

Because of the key regulatory role of the Wnt/β-catenin signalling pathway during the differentiation of Müllerian ducts, we continued activating the Wnt/β-catenin signalling pathway with a 3 μM CHIR99021 treatment for 2 days (CHIR 2d) to preferentially generate Müllerian duct-like cells (concentration and time have been optimised). After induction, the cells were cobblestone-shaped and epithelium-like (Fig. 1b). The expression levels of *KRT8*, *VIM*, and Müllerian duct marker *WNT7A*^19^ were strongly increased, and a specific marker for the multipotent nephron progenitor population *SIX2*^22,27–29^ was significantly downregulated (Fig. 1c), suggesting that CHIR 2d treatment led to IM cell differentiation toward the Müllerian duct, but not renal lineage development. Additionally, immunofluorescence showed that the protein level of PAX2 was positively detected (99.61 ± 0.18%, *N* = 3) in these cells (Fig. 1d and Supplementary Fig. 2a). The expression of KRT8 and VIM in the cells was also stronger than that in the previous IM stage (Fig. 1d), consistent with the results of RNA expression levels. A high positive rate of pan-cytokeratin (pan-CK, including KRT 4, 5, 6, 8, 10, 13 and 18) and VIM was detected in these cells (Fig. 1e and Supplementary Fig. 2b). According to flow cytometry analysis, more than 90% of these differentiated cells were double-positive for KRT8 and VIM (Fig. 1e and Supplementary Fig. 2c). These features of the epithelial/mesenchymal hybrid state are similar to those of the early-stage Müllerian duct^30^.

Furthermore, RNA-seq analysis showed that the upregulated genes after CHIR 2d treatment were IM marker LHX1 (also acts as a marker of a developing Müllerian duct)^24^, epithelial markers (*KRT8* and *KRT18*), and mesenchymal markers (*VIM*, *FBN1*, *FN1*, *MMP11*, *COL1A1* and *PDGFFRA*). GO analysis identified the enrichment of epithelial cell development, cardiac chamber morphogenesis, the response to steroid hormones, and reproductive structure development. These results indicate that the characteristics of cells after CHIR 2d treatment resemble those of the embryonic Müllerian duct; therefore, these cells are defined as MDLCs. These hPSC-derived MDLCs retained their normal karyotype after induction (Fig. 1h). In addition, teratomas were not found in any of the nude mice after receiving MDLC transplantation for 12 weeks (Supplementary Fig. 2d). Hence, the defined chemicals and steps can effectively differentiate hPSCs into MDLCs via the mesendoderm and IM stages.

### 3D culture the hPSC-derived MDLCs to form functional full-thickness endometrial organoids with epithelium–stroma in vitro

**Fig. 2:**
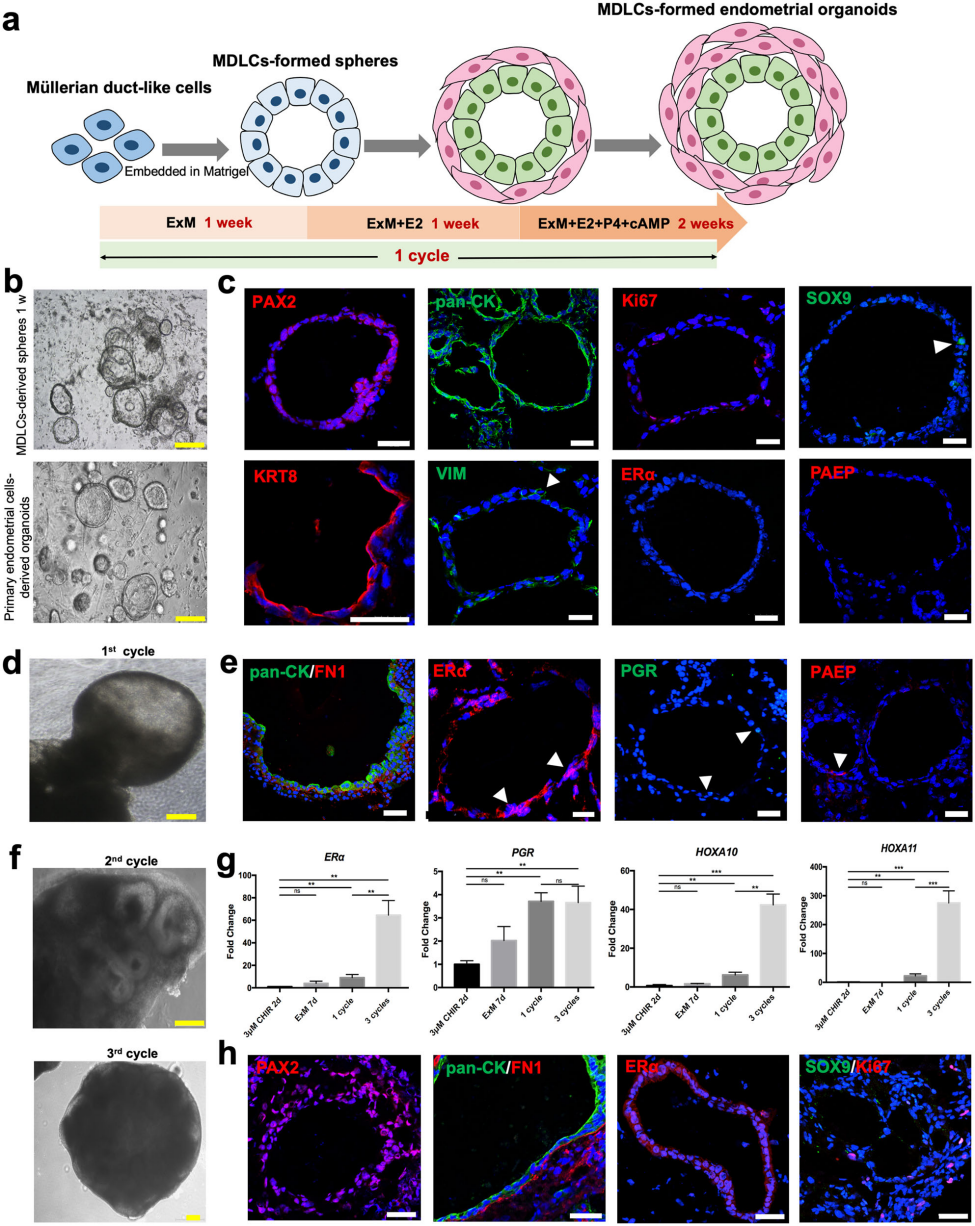
Generation of MDLC-derived full-thickness endometrial organoids with epithelium–stroma in vitro. **a** Schematic of the generation of endometrial organoids from hPSC-derived MDLCs in vitro 3D culture. ExM endometrium organoid expansion media, E2 oestrogen, P4 progesterone, cAMP cyclic adenosine monophosphate. **b** Morphology of MDLC-derived spheres and primary endometrial cell-derived organoids after 3D culture for 1 week. Yellow scale bars, 200 μm. **c** Immunofluorescence of multiple markers (PAX2, pan-CK, KRT8, VIM, Ki67, SOX9, ERα, and PAEP) in MDLC-derived spheres. White scale bars, 40 μm. DAPI (blue). **d** Formation of MDLC-derived endometrial organoid after one cycle culture. Yellow scale bars, 200 μm. **e** Identification of features (pan-CK, FN1, ERα, PGR and PAEP) in MDLC-derived endometrial organoid after 1 cycle culture. White scale bars, 40 μm. DAPI (blue). **f** Morphology of MDLC-derived endometrial organoid after two cycles and three cycles culture. Yellow scale bars, 200 μm. **g** Expression level of marker genes from MDLCs to endometrial organoids. (Data represent mean ± SEM, *N* = 3. *P < 0.05; **P < 0.01; ***P < 0.001; ns, *P* > 0.05 by one-way ANOVA and unpaired, two-tailed Student’s *t* test) **h** Maintenance of features in MDLC-derived endometrial organoid after long-term culture (three cycles). White scale bars, 40 μm. DAPI (blue).

To provide optimal and suitable conditions to mimic the in vivo microenvironment for cell differentiation, we embedded hPSC-derived MDLCs in Matrigel and cultured them with endometrium organoid expansion media (ExM), as reported in Turco’s research^31^ (Fig. 2a). After 1 week, these cells gradually aggregated and grew into hollow spheres, similar to the morphology of primary endometrial epithelial cell-derived organoids (Fig. 2b and Supplementary Fig. 3a). Conversely, no growth of obvious clusters was observed in the group cultured in the control group (DMEM/F12 medium), and clusters inconsistent in size and shape were observed in the serum-cultured group (DMEM/F12 medium containing 10% FBS) (Supplementary Fig. 3b). Immunofluorescence results showed that almost all the cells of the spheres still expressed PAX2 and KRT8 or pan-CK, and most cells were VIM-positive (Fig. 2c). SOX9, a marker of progenitor cells in some tissues, is expressed at the base of endometrial gland cells in vivo^32–34^, and SOX9 + epithelial cells can generate gland-like structures in vitro^31^. In the spheres, SOX9 was also found to be positively expressed in multiple cells, as well as the proliferating marker Ki67, similar to that in primary endometrial epithelial cell-derived organoids (Fig. 2c and Supplementary Fig. 3c). However, mature endometrial markers such as steroid hormone receptor oestrogen receptor α (ERα), progesterone receptor (PGR), and progestagen-associated endometrial protein (PAEP), which are secreted by mature endometrial glandular epithelium^35^, were almost undetectable levels in these spheres (Fig. 2c).

Thus, to further promote the differentiation of these spheres into mature endometrium and express specific markers to enable decidualisation responses, they were sequentially treated with oestrogen (E2), progesterone (P4), and cyclic adenosine monophosphate (cAMP)^31,36^. After one cycle of induction with steroid hormones (ExM for 1 week, ExM + E2 for 1 week, and ExM + E2 + P4 + cAMP for 2 weeks) (Fig. 2d), ERα, PGR, and PAEP were positively expressed in the spheres (Fig. 2e). The expression pattern of these specific markers in hPSC-derived spheres was similar to that in the primary endometrial epithelial cell-derived organoids^31^; therefore, we named these spheres MDLC-formed endometrial organoids. Moreover, immunostaining results revealed that the spheres consisted of epithelial cells (pan-CK-positive) and stromal cells (FN1-positive) (Fig. 2e), demonstrating the bidirectional differentiation potency of MDLCs. The epithelial cells were allowed to form a lumen, and the stromal cells constituted the basal layer (Fig. 2e and Supplementary Fig. 4a), resembling the native structure of full-thickness endometrium, in which the primary endometrial cells cannot form in vitro.

These results demonstrate that hPSC-derived MDLCs can form functional full-thickness endometrial organoids in an in vitro 3D culture system, which consists of an epithelium–stroma structure and responds to steroid hormone treatments.

### Long-term culture of MDLCs-formed endometrial organoids in vitro

To further investigate the properties of MDLC-formed endometrial organoids, we cultured them for another two cycles in ExM with sex hormones. We found that the compactness of organoid spheres increased as the culture time increased, indicating that organoid cells constantly proliferated (Fig. 2d, f). The total mRNA expression of *ERα* and *PGR* significantly increased (Fig. 2g). *HOXA10* and *HOXA11* are expressed in the mesenchyme, playing an important role in the developmental patterning of female reproductive ducts^37,38^; they are also expressed in endometrial stromal cells of adult mice and humans because they are essential for proper endometrium development and normal implantation of embryos^39,40^, and were also detected to have a distinctly higher expression in long-term cultured organoids (Fig. 2g). Immunofluorescence showed that the organoids maintained a full-thickness structure with pan-CK-positive epithelia and FN1-positive stroma (Fig. 2h). In addition, some epithelial and stromal cells of the organoids still expressed the proliferating marker Ki67 and progenitor marker SOX9 (Fig. 2h and Supplementary Fig. 4b). The expression of ERα was confirmed at the protein level; the positive rate of ERα was 55.32 ± 10.48% in epithelial-stromal organoids and 93.05 ± 3.05% in epithelia of the organoids (*N* = 3) (Fig. 2h and Supplementary Fig. 4c). These results indicate that MDLC-formed endometrial organoids can be expanded in long-term 3D culture in vitro and maintain the polarisation structure of epithelia and stroma, with the positive expression of endometrial-specific markers.

### Transplantation of hPSC-derived MDLCs beneath the renal capsule to form endometrium-like tissues

**Fig. 3:**
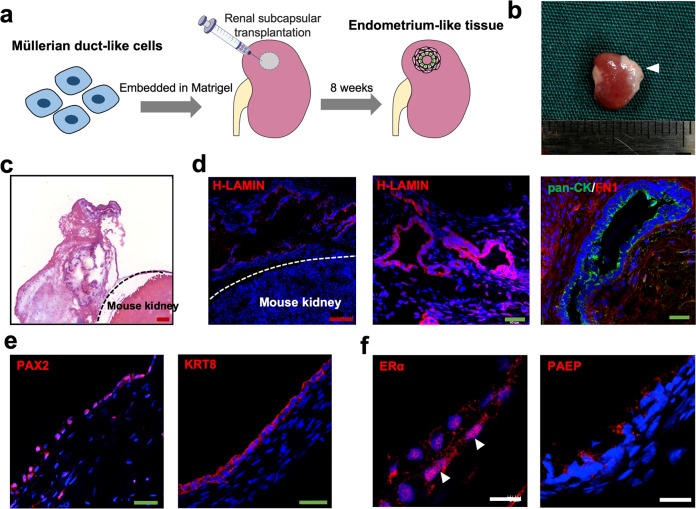
Formation of endometrium-like tissues from hPSC-derived MDLCs in vivo. **a** Schematic of hPSC-derived MDLCs transplanted beneath the renal capsule to form endometrium-like tissues. **b** Macroscopy of the white graft in transplanted site (arrowhead) of nude mice 8 weeks after xenotransplantation. **c** Histological staining of the mass and mouse kidney. Red scale bars, 200 μm. **d** Immunofluorescence for anti-human LAMIN (H-LAMIN) antigen in the mass, the H-LAMIN positive cells contain epithelial and stromal cells. Red scale bars, 200 μm; green scale bars, 40 μm. **e** Expression of PAX2 and KRT8 in the mass. Green scale bars, 40 μm. **f** Identification of specific endometrial makers ERα and PAEP in the mass. White scale bars, 10 μm.

To determine whether hPSC-derived MDLCs could differentiate into endometrial cells in vivo, they were transplanted into the renal capsules of female nude mice (Fig. 3a). After implantation for 8 weeks, a white graft was observed in the renal capsule (Fig. 3b), and microscopic examination showed multiple cystic masses (Fig. 3c). Immunofluorescence studies revealed that some cells in the mass were stained exclusively with anti-human LAMIN (H-LAMIN) antibody located in the lumen and surrounding tissue (Fig. 3d). The lumen cells of the mass positively expressed pan-CK, and the surrounding cells expressed FN1, suggesting that they were epithelial and stromal cells that differentiated from hPSC-derived MDLCs (Fig. 3d). PAX2 was positively detected in epithelia and stroma (Fig. 3e). In addition, positive expressions of ERα and PAEP were detected (Fig. 3f). These results demonstrate that hPSC-derived MDLCs can differentiate into endometrium-like tissues with epithelia-stroma structures after xenotransplantation beneath the renal capsule.

### Evaluation of morphological and histological endometrium parameters after MDLCs repair in full-thickness endometrial injury

**Fig. 4:**
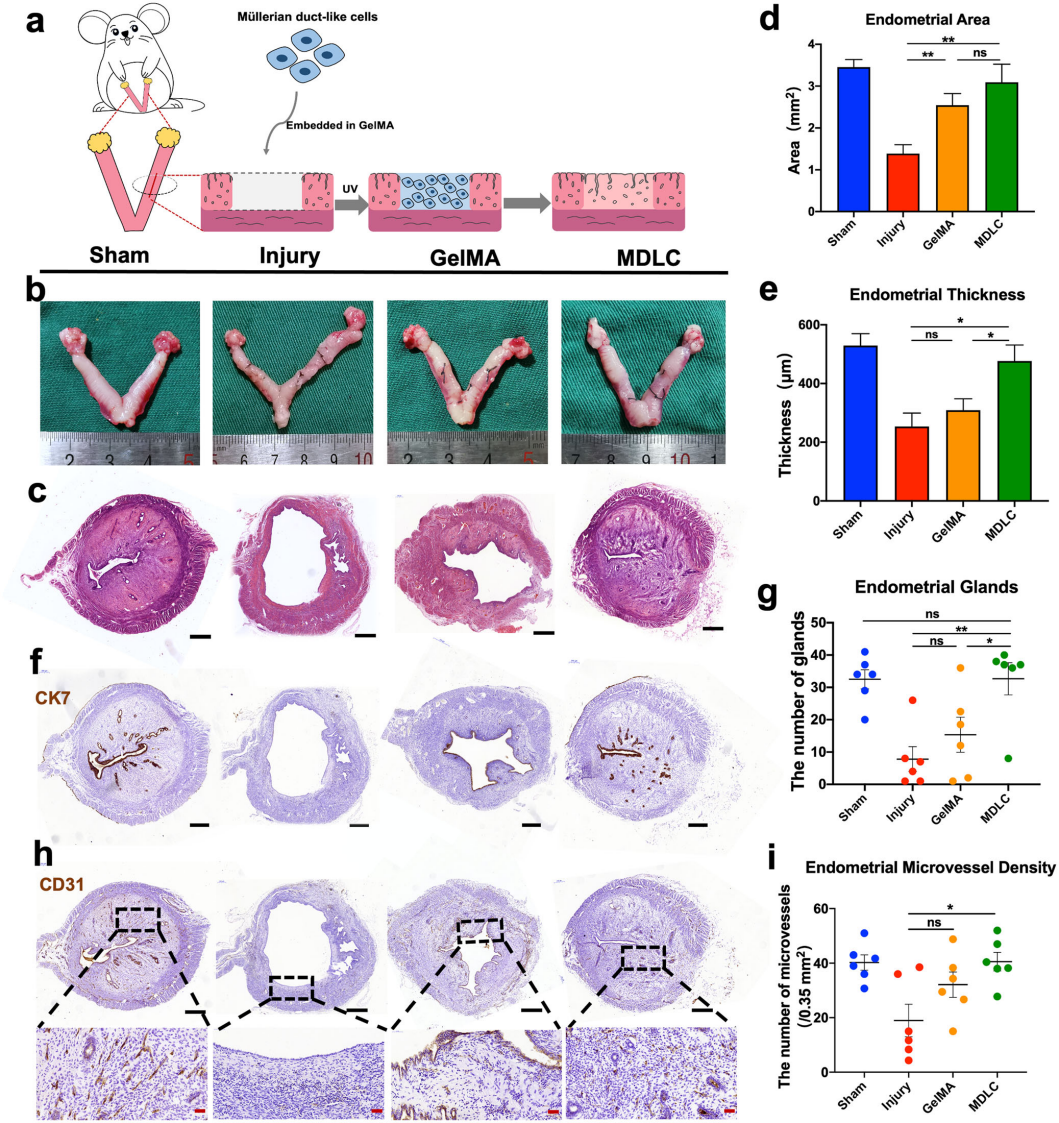
Regenerative effects of hPSC-derived MDLCs in full-thickness endometrial injury. **a** Schematic of hPSC-derived MDLCs transplantation for repairing full-thickness endometrial injury in rat models. **b** Gross appearance of uterine horns in each group after surgery for 4 weeks. **c** Histological staining of the surgical area in each group (Sham, Injury, GelMA and MDLC group). **d** Statistical analysis of the average endometrial area measured at the surgical segment of each group. **e** Statistical analysis of the average endometrial thickness measured at the surgical segment of each group. **f** Immunohistochemistry of the endometrial epithelial maker (CK7) in each group. **g** Statistical analysis of the average endometrial glands at the surgical segment of each group. **h** Immunohistochemistry of the vascular endothelial maker (CD31) in each group. **i** Statistical analysis of the average endometrial microvessel density in each group. Black scale bar, 500 μm; red scale bar, 50 μm. (Data represent mean ± SEM, *N* = 6 uterine horns per group. *P < 0.05; **P < 0.01; ns, *P* > 0.05 by one-way ANOVA and unpaired, two-tailed Student’s *t* test.).

hPSC-derived MDLCs have the potential to differentiate into endometrial lineage cells consisting of both epithelial and stromal cells in vitro and in vivo. To assay the capacity to repair the full-thickness endometrial injury in rat models, MDLCs were embedded in gelatin methacrylate (GelMA) hydrogel^41^ (which displayed good biocompatibility for MDLCs; Supplementary Fig. 5), fully distributed to fill the endometrial defect site with the capacity of fluidity, and rapidly solidified under UV irradiation to form a ‘wound dressing’ (Fig. 4a). After 4 weeks, the gross appearance showed that the size of the uterine horns in the GelMA group (only GelMA for repair after full-thickness endometrial injury) and MDLC group (MDLCs loaded with GelMA for repair) were relatively uniform and that there were fine vasculatures in the surgical segment, without differences from those in the sham group. However, the uterine horns in the injury group (spontaneous repair after full-thickness endometrial injury) were slightly collapsed and had decreased vasculature (Fig. 4b).

Histological staining revealed that the surgical segment in each group had a complete cavity surface consisting of a monolayer of epithelial cells (Fig. 4c). Both the MDLC and GelMA groups showed the formation of new endometrial tissue with a glandular structure; the injury group had a thin endometrial wall but neither new tissue nor glands. The measurement results showed that the total endometrial area of the MDLC group (3.09 ± 0.43 mm^2^) and GelMA group (2.54 ± 0.28 mm^2^) was significantly larger than that of the injury group (1.39 ± 0.21 mm^2^) (*N* = 6, *P* < 0.01) (Fig. 4d). And the average endometrial thickness of the MDLC group (476.80 ± 54.26 μm) was significantly thicker than that of the GelMA group (309.00 ± 38.84 μm) and injury group (253.60 ± 45.52 μm) (*N* = 6, *P* < 0.05) (Fig. 4e).

Cytokeratin 7 (CK7)^13,31^ immunohistochemical staining was performed to observe endometrial gland formation (Fig. 4f). The statistical results showed that the number of glands in the MDLC group (32.67 ± 4.96) was higher than that in the injury group (7.83 ± 3.83) and GelMA group (15.33 ± 5.43) (*N* = 6, *P* < 0.01), nearly reaching the number in the sham group (32.50 ± 2.9) (Fig. 4g).

CD31 immunohistochemical staining was performed to observe the endometrial microvessel formation in each group (Fig. 4h). The statistical results showed that microvessel density in the MDLC group (40.56 ± 3.41) was significantly higher than that in the injury group (18.98 ± 5.97) (*N* = 6, *P* < 0.05) and close to that in the sham group. But there was no significant difference between GelMA group (32.10 ± 4.66) and injury group (*P* > 0.05) (Fig. 4i).

Overall, the MDLC group showed good regenerative effects with more native physiological structure reconstruction via assessment of the total endometrial area, average endometrial thickness, gland formation, and neovascularisation.

### Cell tracing and scRNA-Seq to reveal the cell fate of hPSC-derived MDLCs in situ repair

**Fig. 5:**
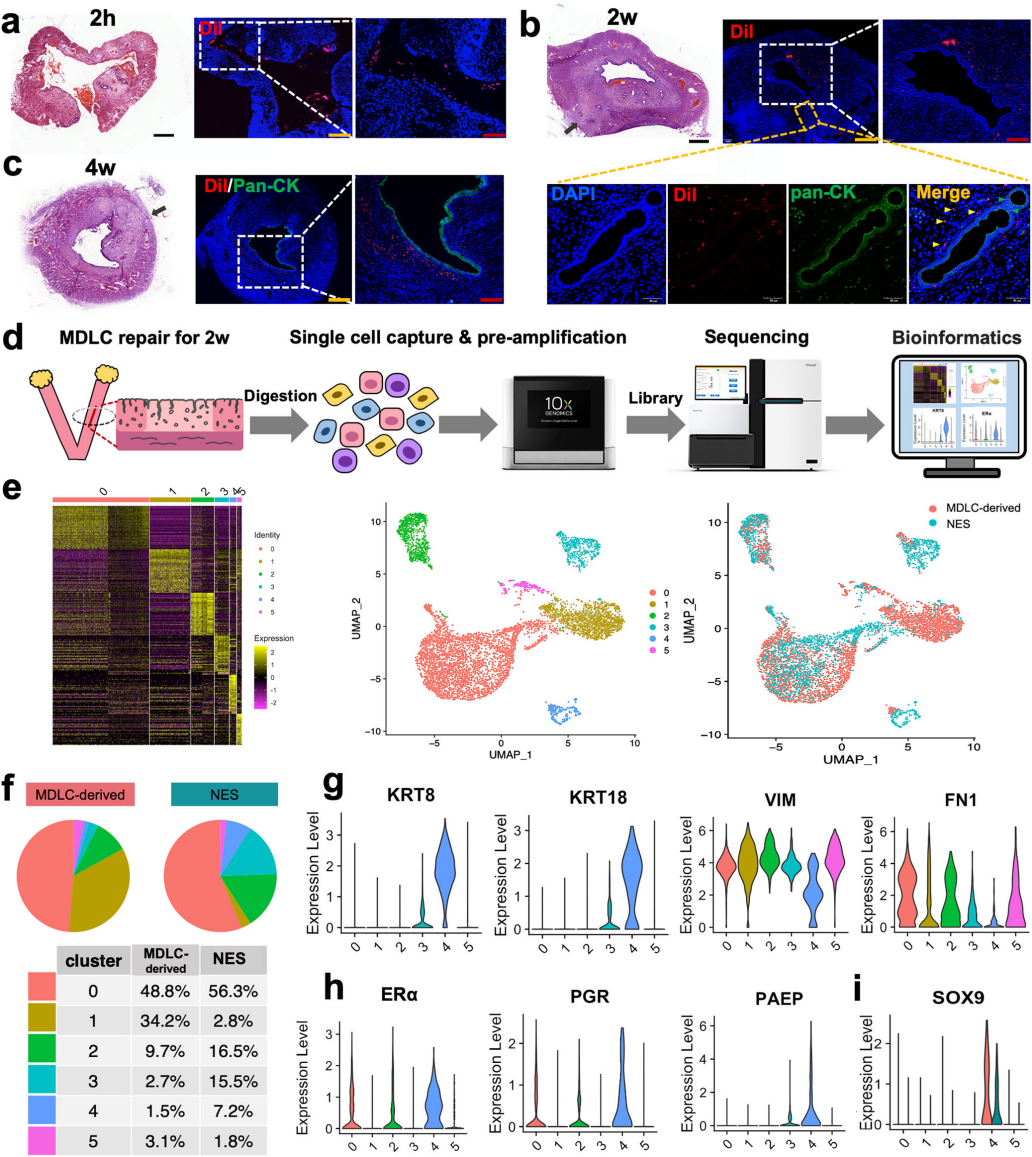
Cell fate tracing and scRNA-Seq analysis of hPSC-derived MDLCs in situ repair. **a**–**c** Tracing of transplanted hPSC-derived MDLCs in repairing the full-thickness endometrial injury at 2 h, 2 weeks, and 4 weeks after surgery. Black/yellow scale bars, 500 μm; red scale bars, 200 μm; white scale bars, 50 μm. **d** Schematic of scRNA-Seq to investigate the fate of transplanted hPSC-derived MDLCs in situ repair after 2 weeks. **e** Heatmap and UMAP showing cell populations in MDLC-derived cells and human normal endometrium in secretory phase (NES), identified by clustering similar single-cell transcriptomes. **f** Cell proportion of MDLC-derived cells or NES in each cluster. **g**, **h** Violin plots of expression levels for epithelial markers (KRT8, KRT18), stromal markers (VIM, FN1), and endometrial-specific markers (ERα, PGR, and PAEP) in MDLC-derived cells. **i** Violin plots of expression levels for SOX9 in cluster 4 from MDLC-derived cells, compared with those of NES.

To investigate the fate of MDLCs in repairing the full-thickness endometrial injury, MDLCs labelled with DiI were loaded into GelMA to repair defects. DiI-labelled cells were found at the edge of the injured endometrial cavity 2 h after transplantation (Fig. 5a). After 2 weeks, DiI-labelled cells were detected in the reconstructed endometrium, and some of them were stained with pan-CK antibody (Fig. 5b), indicating that MDLCs may contribute to differentiation into both epithelial and stromal cells. Although DiI-labelled cells were mostly observed in the stroma after 4 weeks (Fig. 5c), some human-derived cells were detected in the epithelium (H-LAMIN + pan-CK + co-expressed) after 6 weeks (Supplementary Fig. 6a). Next, scRNA-Seq was performed to dissect the fate of MDLC differentiation after endometrial repair for 2 weeks (Fig. 5d). A total of 4022 human-derived cells were captured, integrated, and comparatively analysed with the normal human endometrium in the secretory phase (NES) dataset (public database of the Genome Sequence Archive for Humans under the accession number HRA000928)^42^. Hierarchical clustering and scRNA-seq data analysis with uniform manifold approximation and projection (UMAP) identified six cell clusters (clusters 0–5) (Fig. 5e), and each cluster contained both MDLCs and NES cells, indicating that the transcriptomic profiles of MDLC-derived cells were similar to those of human endometrial cells in the secretory phase in vivo (Fig. 5e, f). The proportion of cells in each cluster differed between MDLC-derived cells and NES cells (Fig. 5f). Violin plots highlight the selection of specific marker expression in the MDLC-derived cells of each cluster. The epithelial markers *KRT8* and *KRT18* were highly expressed in cluster 4 epithelial cells. The stromal markers *VIM* and *FN1* were high in other clusters, especially in clusters 0, 2 and 5, suggesting that these cells were committed to the stroma. (Fig. 5g and Supplementary Fig. 5b). In addition, the endometrial markers, including *ERα*, *PGR* and *PAEP*, in MDLC-derived cells of cluster 4 were more highly expressed than the others, but the levels of *PGR* and *PAEP* were lower than those of NES cells (Fig. 5h and Supplementary Fig. 5c). In addition, the progenitor marker *SOX9* was highly expressed in both MDLC-derived cells and NES of cluster 4, suggesting the capacity to maintain the ‘stemness’ of the endometrial epithelium in vivo (Fig. 5i). These findings reveal the fate of hPSC-derived MDLCs in the in situ repair of full-thickness endometrial injury in a rat model, which can differentiate into epithelial and stromal cells, similar to human normal endometrium.

### Pregnancy function of the reconstructed endometrium after hPSC-derived MDLCs repair

**Fig. 6:**
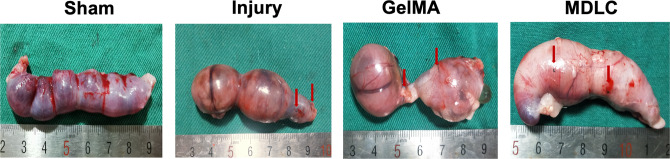
Pregnancy outcome improvement after MDLC treatment. Representative images of a pregnant uterus in each group prior to birth (red arrow/marking sutures indicate surgical sites).

The goal of endometrial repair is to provide fertile ‘soil’ for embryo implantation, growth and development. To investigate the functionality of hPSC-derived MDLCs in endometrial repair, we conducted reproductive studies after repairing full-thickness endometrial injuries for 12 weeks. Pregnancy was observed in some of the regenerative uterine horns (Fig. 6), and the outcomes are shown in Table 1. The pregnancy rate of the sham group (91.67%) (*P* < 0.05) and the MDLC group (83.33%) (*P* < 0.05) was significantly increased, and there was no significant difference for GelMA group (58.33%), when compared with that of injury group (50%) (Table 1). Most embryos were implanted in normal areas rather than at surgical sites; however, embryos were found at surgical sites of the MDLC group, with a proportion of 3/10 pregnancy uterine horns, which was 1/7 in the GelMA group (Table 1). Although the average gestational sacs of the MDLC group (2.3 ± 0.4) were lower than those in the sham group (5.8 ± 0.7) (Table 1), these findings suggest that the endometrial functional recovery of the MDLC group was better than that of the injury and GelMA groups.

**Table 1:** Reproductive outcomes in experimental rats (*N* indicates the number of uterine horns).

Groups outcomes	Sham, *N* = 12	Injury, *N* = 12	GelMA, *N* = 12	MDLC, *N* = 12	*P* value
Pregnancy rate (%)	91.67 (11/12)^*^	50 (6/12)	58.33 (7/12)	83.33 (10/12)^†^	*P* < 0.05^‡^
Pregnancy at surgical site	/	0	1	3	
Gestational sac	5.8 ± 0.7	1.3 ± 0.4	1.3 ± 0.4	2.3 ± 0.4^§^	*P* ≤ 0.0001^||^

*P < 0.05, Sham group versus Injury group; ^†^*P* < 0.05, MDLC group versus Injury group; ^‡^Chi-square test; ^§^*P* < 0.05, MDLC group versus Injury group; ^||^Kruskal–Wallis test. Data are presented as mean ± SEM.

## Discussion

Stem cell-based therapy plays a core role in regenerative medicine, and hPSC-derived cell-based strategies have opened new frontiers in medicine with the possibility of regenerating lost or damaged cells^43^. In this study, hPSCs were induced to differentiate into MDLCs using the developed protocol. hPSC-derived MDLCs possess the ability of bipotential endometrial lineage differentiation, which can further differentiate and form endometrial organoids with endometrial epithelial and stromal cells in vitro and in vivo. In addition, hPSC-derived MDLCs showed good therapeutic effects on structural and functional recovery in a full-thickness endometrial injury rat model.

hPSC-derived MDLCs possess the characteristics of endometrial lineage and bipotential to further differentiate into endometrial epithelial cells and stromal cells, which are regarded as a new type of seed cells to simultaneously repair both endometrial epithelial cells and stromal cells. Studies have reported that the epithelial/mesenchymal hybrid state showed remarkable plasticity and positively correlated with stemness in multiple organs during organogenesis^30,44^, suggesting that MDLCs are intermediate mesoderm-derived precursors with positive co-expression with PAX2, KRT8 and VIM. MDLCs can specifically differentiate and form endometrial organoids/tissues in vitro in 3D culture and in vivo. The differentiated endometrial cells consisted of both endometrial epithelial cells and stromal cells, indicating the bi-potent differentiation of MDLCs. Stem cell therapy for endometrial diseases has limitations. On the one hand, the sources of most seed cells are of limited supply, and they have a finite capacity to proliferate. Specific lineage differentiation of these seed cells into the endometrium is also challenging. Zhang et al. found that mouse BMSCs had the potential to differentiate into endometrial epithelial cells when co-cultured with endometrial stromal cells in vitro^45^. Jing et al. showed that rat BMSCs could only differentiate into endometrial stromal cells^46^. Ong et al. claimed that BMSCs do not contribute to endometrial cell lineages^14^. Thus, the endometrial-specific differentiation of BMSCs and other mesenchymal cells remains debated. Overall, the obtained MDLCs are abundant because of the unlimited proliferative capacity of hPSCs and their endometrial-specific and bipotential differentiation abilities, which can be applied as the recommended seed cells for the repair and regeneration of endometrial injury in clinical settings.

The generation of hPSC-derived MDLCs indicates that the workflow of induction with well-defined chemicals and steps is simple and effective. Several studies have reported that hPSCs can differentiate into endometrial cells. Parasar et al. reported that hESCs spontaneously differentiated in vitro, and the expression of multiple genes related to endometrial development, endometrial structure, and endometrial cells was detected after differentiation^47^. Ye et al. induced the differentiation of hESCs into the Müllerian duct epithelium by co-culturing with undifferentiated mouse neonatal uterine mesenchyme^48^. And Jiang et al. recently obtained endometrial membrane organoids from hESCs accompanied by human endometrial stromal cells in an in vitro 3D culture system^49^. However, neither spontaneous differentiation nor co-culture is suitable for the large-scale generation of endometrial cells, because of the undirectional and low-efficiency differentiation from hPSCs. The critical first step in directing the differentiation of hPSCs into endometrial cells is to emulate the developmental stages of the Müllerian duct during embryogenesis. Miyazaki et al. directed the differentiation of hiPSCs through the IM, coelomic epithelium, and Müllerian duct to endometrial stromal fibroblasts^50^ under molecularly defined embryoid body (EB) culture conditions^51^, but this method still cannot achieve the simple, rapid generation of endometrial cells. Cheung et al. described a monolayer differentiation protocol for inducing hiPSCs through MD mesenchyme to generate large numbers of highly purified human endometrial stromal fibroblasts^52^, which established a useful method as a demo. Similarly, we found appropriate developmental signals that simulate the development of the early embryonic Müllerian duct and induced hPSCs through the mesendoderm and IM to the Müllerian duct stage with defined compounds and growth factors in the 2D environment. In this study, we propose a simple, clear, and efficient differentiation protocol for MDLCs from hPSCs.

hPSC-derived MDLCs have a significant repair effect on full-thickness endometrial injury in rats. After the repair of MDLCs, the structure of the damaged endometrium can be reconstructed by increasing the area and thickness of the endometrium and promoting the formation of glands and microvessels. These factors are required to regulate endometrial receptivity during embryo implantation^53^. The results of the pregnancy test demonstrated that MDLCs restored pregnancy function. Compared with the injury group and GelMA group, the pregnancy rate and the number of pregnancy at the surgical site increased in the MDLC group. Few studies have attempted the application of hPSC-derived cells in uterine regeneration. However, because there are few reliable differentiation protocols for hPSCs^16,49^ or hPSC-derived cells, replenishing both epithelial and stromal cells in repairing full-thickness injury of the endometrium is difficult^17^. Our hPSC-derived MDLCs play an important role in full-thickness endometrial injury in rats, realising both structural and functional repair.

DiI labelling and scRNA-Seq were used to trace the cell fate of MDLCs and reveal the principle of MDLCs in situ repair of the endometrium. DiI-labelled cells appeared in the uterus of rats, indicating that MDLC-derived cells participated in the formation of endometrial structures after implantation. After 2 weeks and 6 weeks, DiI-labelled cells were detected both in endometrial epithelia and stroma, reflecting the bi-potency of MDLCs, which can differentiate into endometrial epithelial cells and stromal cells in vivo. The scRNA-Seq analysis demonstrated that in situ repair of MDLC-derived cells for 2 weeks indicates that the transcriptome of MDLC-derived cells is similar to that of human endometrial cells, and MDLCs can differentiate into specific endometrial epithelial and stromal cell lineages. In addition, MDLC-derived cells in clusters 1 and 3 were found to have a high expression of *vascular endothelial growth factor A* (*VEGFA*) (Supplementary Fig. 6d), which plays a role in vascularisation^54^. MDLC-derived cells in cluster 3 expressed the vascular marker *vWF*, but the level was significantly lower than that in cluster 3 (Supplementary Fig. 6d). Even though, it showed that MDLC-derived cells may be regulated to differentiate into endothelial cells and promote the reconstruction of endometrial microvessels in the microenvironment of endometrial injury.

Our study provides an easily accessible cell source for hPSC differentiation with a simple, effective protocol and a new treatment strategy for endometrial regeneration, which has pre-clinical implications. In addition, it provides a full-thickness endometrial organoid model for in vitro studies, such as drug screening and toxicity tests for the female reproductive system. However, there are still some limitations in this study. The optimisation and purification of MDLCs should be considered to improve the efficiency and safety of cell therapy, and this treatment strategy should be applied in large animal experiments to further verify its effectiveness. In addition, hiPSCs might be an appropriate choice which can be reprogrammed from patient adult cells for clinical use, considering the limited cell sources, ethical issues of hESCs, and immunogenicity of allogeneic cells.

## Methods

### Animals

All animal experiment protocols were approved by the ethical committee of the school of medicine, Zhejiang university (ZJU2018094) and were in compliance with institutional guidelines. Two kinds of animals were utilised in our study: female nude mice for teratomas forming assay and in vivo transplantation beneath the renal capsule; rats for investigating the repair effects of hPSCs-derived MDLCs. All animals were purchased from the Shanghai SLAC laboratory animal Co., Ltd, and housed at the Zhejiang university animal facility.

### hPSCs culture for maintenance and differentiation

Three hPSCs cell lines, including hUiPSCs (human urine-derived induced pluripotent stem cells, a gift from professor Junfeng Ji’s lab)^55,56^, hMiPSCs (human monocytes-derived induced pluripotent stem cells, a gift from professor Yanxin Li’s lab)^57,58^ and hESC h9 (WiCell), were cultured on plates with coated Matrigel (Coring), and maintained in mTeSR1 media (Stem Cell). Cells were passaged at a ratio of 1:3–1:6 every 6–8 days using ReleSR dissociation solution (Stem Cell). For IM cell differentiation, hPSCs were sequentially treated with 5 μM CHIR99021 (Selleck) in RPIM 1640 medium (Gibco) containing 1% antibiotics (50 U/ml penicillin and 50 mg/ml streptomycin) (Gibco) for 36 h into mesendodermal cells; then 100 ng/ml basic fibroblast growth factor (bFGF) (Peprotech) and 10 nM Retinoic acid (RA) (Sigma-Aldrich) in RPIM 1640 medium containing 1% antibiotics (50 U/ml penicillin and 50 mg/ml streptomycin) were used to induce efficient differentiation of hPSCs into IM cells. For MDLCs differentiation, the IM cells were then treated with 3 μM CHIR99021 + 1% antibiotics + RPIM 1640 medium for 2 days. The treatments of cell lines were in accordance with the Declaration of Helsinki.

### 3D culture and hormonally stimulation of hPSCs-derived MDLCs to form endometrial organoids in vitro

hPSCs-derived MDLCs were dissociated with ReleSR solution for 8 min in 37 °C incubator, centrifuged, and resuspended at a concentration of 5 × 10^6^ cells/ml in DMEM/F12 medium (Gibco), mixed with Growth Factor Reduced Matrigel (Corning) at a ratio of 1:1 (vol:vol), dripped 20 μl/drop on the low adhesion plate (Corning), after gelatinisation at 37 °C for 30 min, added endometrium expansion medium (ExM)^31^ and gently scraped drops to 3D suspension culture. After 1 week, cell aggregates spheres were primed stimulated with 10 nM E2 (β-oestradiol, Sigma) in ExM for an additional of 1 week. Then the medium was replaced with 10 nM E2 + 1 μM P4 (progesterone, Sigma) + 1 μM cAMP (8-bromoadenosine 3’,5’-cyclic monophosphate, Sigma) in ExM and cultured for another 2 weeks in order to induced organoid maturation. The culture was continued for another two cycles (1 cycle: ExM for 1 week + ExM + E2 for 1 week + ExM + E2 + P4 + cAMP for 2 weeks) to evaluate organoids in long-term culture. The cell source of primary endometrial cells-derived organoids was from the non-pathological part of endometrium from hysterectomy due to leiomyoma in patient, which was approved by the ethical committee of the first affiliated hospital, school of medicine, Zhejiang university (ethics approval No. 2018-113)^42^.

### RNA extraction, reverse transcription, real-time quantitative polymerase chain reaction (qPCR)

Total RNAs were extracted from hPSCs, mesendodermal cells, IM cells, MDLCs and endometrium organoids using Trizol Reagent (Toyobo). The concentration of total RNA was measured by NanoDrop (Thermo Fisher Scientific). Each sample was reverse-transcribed using the Reverse transcription reagent (Toyobo) to produce cDNA. The cDNA was quantified by real-time qPCR using SYBR Green qPCR Master Mix (Takara) and CFX96 Real-Time PCR Detection System (Bio-Rad). Quantification of the samples was performed according to the threshold cycle using the ΔΔCt method. These experiments were repeated three times. The primers are listed in Supplementary Table 1.

### RNA-seq

RNA-seq was modified from a previous method^59^. Briefly, RNA was extracted from samples by Trizol reagent (Takara), reverse transcription was conducted by SuperScript II reverse transcriptase (Invitrogen), and then the obtained double-strand cDNA was conducted using NEBNext mRNA second strand synthesis kit^60^. Subsequently, the double-strand DNA was cleaned with AMPure XP beads (Beckman Coulter), sequencing library was constructed with Nextera XT kit (Illumina) and sequenced on Illumina X10 platform. The raw data were uploaded in public database of the Genome Sequence Archive for Human (HRA002268). Expression levels were calculated with counts per million (CPM) and analysed with DESeq2^61^, differential expressed genes were selected with *P* value <0.05. Gene ontology analysis was performed using Gene Ontology Consortium (http://www.geneontology.org) (Version 2021-10-26, 10.5281/zenodo.5608599).

### Xenotransplantation of hPSCs-derived MDLCs beneath the renal capsule

Female nude mice were anaesthetised by intraperitoneal injection of 8 mg/kg sodium pentobarbital (Sigma) (adjust doses according to weight). The left kidney was exteriorised through a dorsal-horizontal incision. A glass capillary tube with a cone-shaped tip was penetrated into the renal capsule^62^. Subsequently, the MDLCs suspension, which was mixed with the Growth Factor Reduced Matrigel (Coring) at a ratio of 1:1, was slowly injected into the opening of the renal capsule by the indwelling needle. The kidney was carefully placed back into the abdominal cavity, then the incision was sewn up. Grafts were harvested after transplantation at 8 weeks.

### Histological section, immunofluorescence, immunochemistry and microscopy imaging

The organoids, grafts beneath the renal capsule and uterus of rats were harvested and fixed in 4% paraformaldehyde for 24 h. Then these samples were dehydrated and embedded in OCT compound (Thermo NEG50), frozen and serially sectioned at 10–14 μm using a Cryostat Microtome (Thermo), then transferred to microscopic slides. For morphological observation, slides were subjected to hematoxylin and eosin (H&E) staining. For immunofluorescence, the samples were permeabilized with 0.3% Triton X-100 in PBS for 10 min. After blocking with 5% BSA for 30 min, they were incubated with primary antibodies listed in Supplementary Table 2 overnight at 4 °C then washed three times for 5 min. They were then incubated with secondary antibodies and nuclear staining (DAPI) at room temperature for 2 h. At last, slides were washed and mounted with Antifade Mounting Medium (Millipore). For immunochemistry, samples were performed heat-mediated antigen retrieval, permeabilized with 0.3% Triton X-100 and, immersed with 3% H_2_O_2_ to inactivate endogenous peroxidases and blocked with 5% BSA. And they were incubated with the primary antibody overnight at 4 °C then the secondary antibody goat anti-rabbit IgG H&L (HRP) (Jackson) for 2 h at room temperature, followed by substrate deposition with DAB horseradish peroxidase colour development kit (Beyotime). Nuclear counterstain was performed with Hematoxylin and followed by mounting. H&E staining and immunochemistry samples were scanned using the slice digital scanner (3DHISTECH). Immunofluorescence staining were imaged and processed by Olympus FV3000 Laser scanning confocal microscope system (Olympus).

### Establishment of full-thickness endometrium injury model in rats and MDLCs transplantation for repair

A total of 72 uterus horns of Sprague Dawley (SD) female rats (8-week-old) were randomly divided into four groups: sham group (*N* = 18), injury group (*N* = 18), gelatin methacrylate (GelMA) group (*N* = 18), and MDLC group (*N* = 18). After anaesthesia, a longitudinal incision was made in the midline of the lower abdomen to expose the uterus. Sham group: only exposed the uterus for 20 min after laparotomy, without any damage to the uterus. Injury group: a longitudinal incision (1 cm) was made on the opposite side of the mesometrium, and the full-thickness endometrium (1 cm × 0.4 cm × 0.1 cm) was gently avulsed with forceps. GelMA group: GelMA hydrogel (~15 μl) was dripped into the wound of each uterus horn after avulsion, and UV irradiation for 15 s was used to crosslink it to confirm that the gel was solid. MDLC group: After injury, hUiPSC-derived MDLCs (5 × 10^5^) loaded with GelMA hydrogel (~15 μl) was dripped into the wound (MDLCs should be labelled with DiI beforehand for cell tracing), and solidified under UV irradiation. The uterine incision was closed with suture and marked at both ends of the uterine surgical site. And the abdominal wall, abdominal skin and fascia were subsequently sutured. Penicillin was used to reduced postoperative infection, and cyclophosphamide (Baxter) was used to reduce implantation rejection of human-derived cells in rats before and after surgery. For morphological and histological evaluation, rats of each group were sacrificed during oestrus and collected the uterus horns at 4 weeks after surgery. For pregnancy function test, the female rats were naturally mated with fertile male rats at 12 weeks after surgery. The female rats were euthanized at 15~19 days after the presence of a vaginal plug which indicating the fertilisation, and uterine horns were examined for the number and site of embryos.

### Single-cell RNA sequencing and data analysis of hPSCs-derived MDLCs in situ repair

After hPSC-derived MDLCs in situ repair for 2 weeks, the surgical segments of uterine horns were cut into fragments, and digested with digestive enzymes mixture of 1.2 U/mL Dispase II (Roche) and 0.2% collagenase I (Sigma) in DMEM medium for 30 min at 37 °C, and neutralised with 10% FBS in DMEM medium. The cell suspension was collected by passing through a 70-μm cell strainer (Corning), centrifugated and added red blood cell lysate to resuspend for 30 min on ice. Then Single-cell capture and pre-amplification were conducted onto the 10X Chromium Controller (10X Genomics) according to the manufacturer’s instructions (Chromium™ Single Cell 3’ Reagent Kit v3). The generated library was sequenced on Illumina X10 platform to achieve an average of 55,000 reads per cell. Sequencing reads were aligned to the human reference genome GRCh38 using the Cell Ranger suite with default parameters. And the data (HRA002267) was integrated and comparatively analysed with the human normal endometrium in secretory phase (NES) dataset (public database of the Genome Sequence Archive for Human under the accession of HRA000928)^42^. Hierarchical clustering and violin plots were generated in Seurat (http://satijalab.org/seurat/)^63^, and UMAP was performed to visualise cells in a two-dimensional space. Cell annotation was performed by assessing the relative expression of specific markers.

### Statistical analysis

The histological endometrium parameters were statistically analysed using Image-Pro Plus 6.0 software. All data were represented as mean ± SEM. One-way ANOVA and unpaired two-tailed Student’s *t* tests were completed using GraphPad Prism 8. The rate was compared with the Chi-square test. *P* values of statistical significance were represented as ***P < 0.001, **P < 0.01, *P < 0.05.

## Supplementary information

Supplementary Materials

## Data Availability

The raw data of RNA-seq and single-cell RNA sequencing are uploaded in the public database of the Genome Sequence Archive for Human (accession code: HRA002268, HRA002267). The remaining main data are available within the main text or the supplementary materials. Additional data inquiries could be addressed to the corresponding author (zouxiaohui@zju.edu.cn).
