# Supplementary material for: Bi-potential hPSC-derived Müllerian duct-like cells for full-thickness and functional endometrium regeneration
Source: NPJ Regen Med. 2022 Nov 23;7:68. doi: 10.1038/s41536-022-00263-2 (PMC9684429; doi:10.1038/s41536-022-00263-2)
Supplement: Supplementary file 1 — Supplementary Materials [file 41536_2022_263_MOESM1_ESM.pdf]

Supplementary Materials for

Bi-potential hPSCs-derived Müllerian Duct-like Cells for Full-thickness and Functional Endometrium Regeneration

Lin Gong, Nanfang Nie, Xilin Shen, Jingwei Zhang, Yu Li, Yixiao Liu, Jiaqi Xu, Wei Jiang, Yanshan Liu, Hua Liu, Bingbing Wu, XiaoHui Zou*

*Corresponding author. Email: zouxiaohui@zju.edu.cn

This PDF file includes:

Supplementary Figure 1-6

Supplementary Table 1-2

Code used for bulk RNA-seq and scRNA-seq analysis

Supplementary Figure 1.

(a) mRNA levels of 7 marker genes showed the expression changes from hPSCs to IM cells. **(b)** Principal component analysis (PCA) of transcriptome from hPSCs to IM cells. **(c) (d)** The heatmap and GO analysis of mesendoderm cells/ CHIR 36h compared to hUiPSCs. Red highlighting, upregulated genes; green highlighting, downregulated genes. **(e) (f)** The heatmap and GO analysis of IM cells/ bFGF+RA compared to mesendoderm cells/ CHIR 36h. Red highlighting, upregulated genes; green highlighting, downregulated genes. (P value < 0.05)

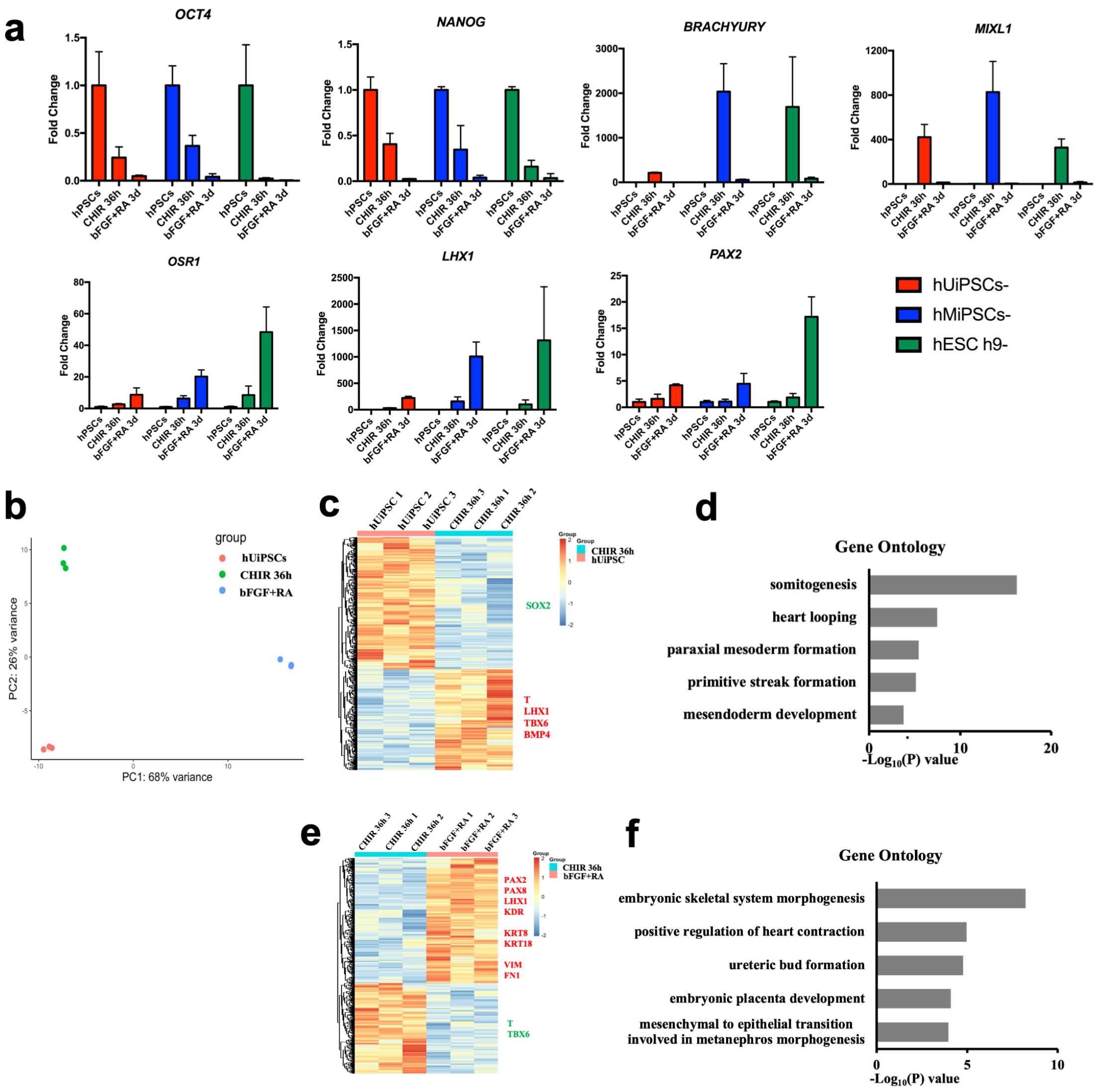

Supplementary Figure 2.

(a) The immunofluorescence of PAX2 in cells after CHIR 2d induction for analyzing the positive rate. (N = 3). **(b)** The expression of pan-CK, PAX2 and VIM in in cells after CHIR 2d induction. DAPI (blue). **(c)** Gating strategy to sort VIM+KRT8+ cells after CHIR 2d induction presented on Fig. 1e. **(d)** No teratomas were found in any of the nude mice after receiving MDLCs transplants for 12 weeks.

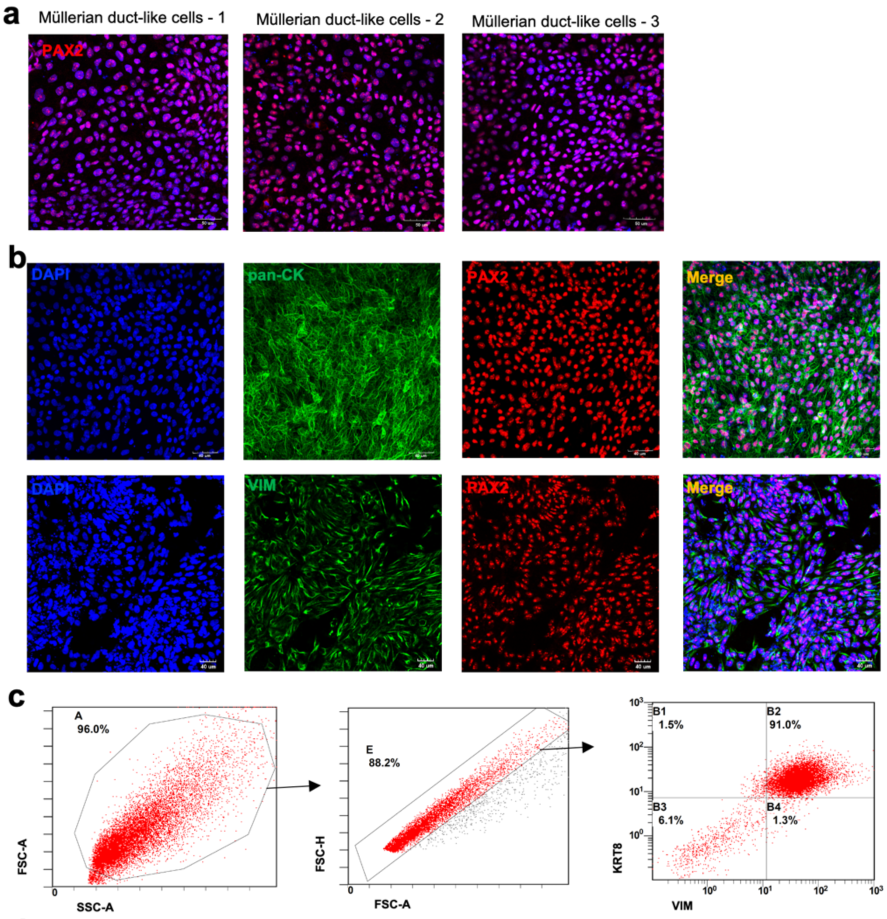

d

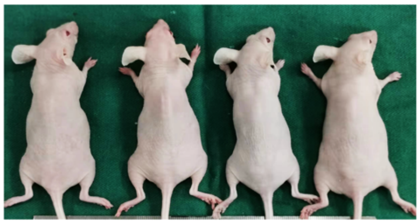

Summary of teratomas forming assay

Cell type	Cell injected	Mice bearing teratoma/total mice injected	Period (week)
hESC h9	$1x10^6$	3/3	6-8
MDLCs	$2x10^6$	0/8	12

Supplementary Figure 3.

(a) The morphology of hPSCs-drived MDLCs in 3D culture with ExM at day 1 and day 3. **(b)** The morphology of hPSCs-drived MDLCs cultured in control group (DMEM / F12 medium) and the serum-cultured group (DMEM / F12 medium containing 10% FBS) at day 7. **(c)** The immunofluorescence of Ki67 and SOX9 in hiPSCs-derived endometrial spheres and primary endometrial cells-derived organoids.

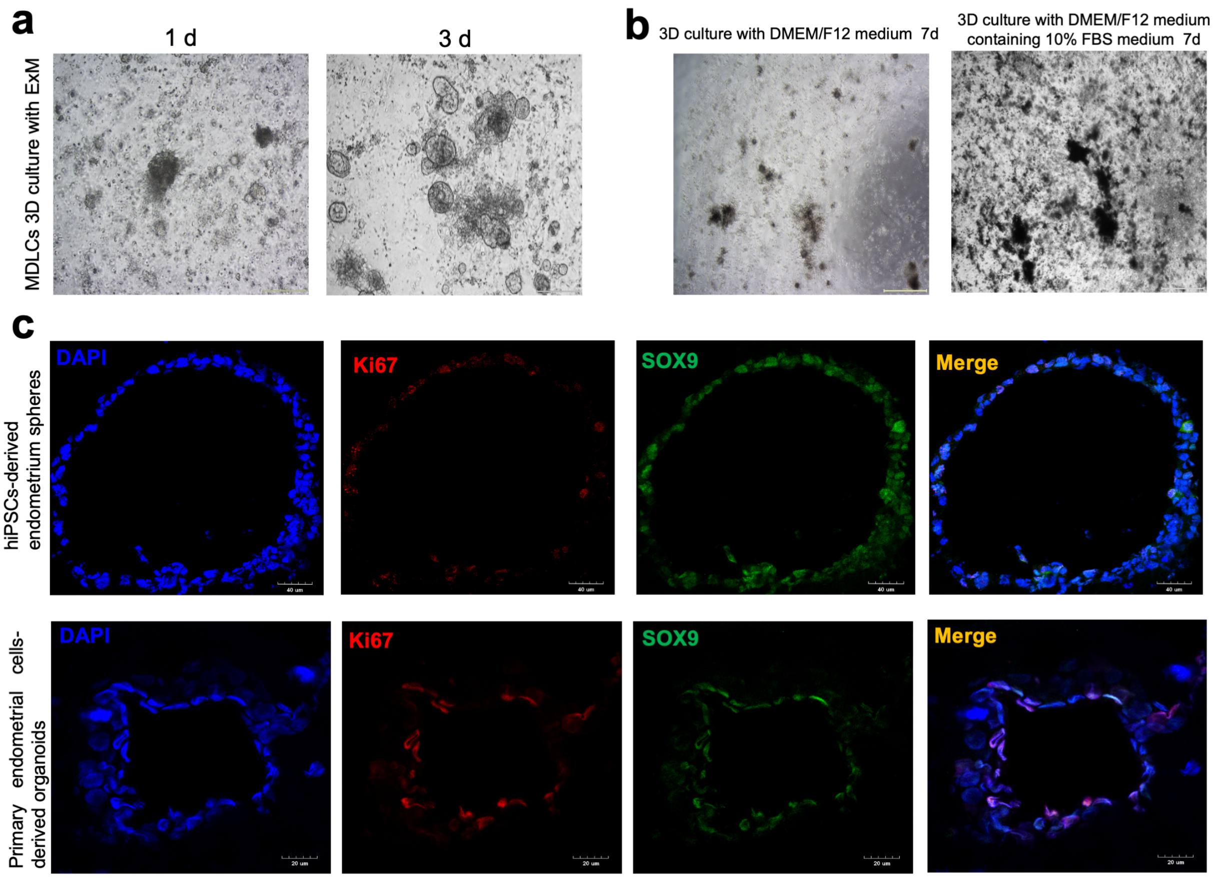

Supplementary Figure 4.

(a) The characterization of specific makers in MDLCs-derived organoids after 1 cycle culture. Scale bars, 40 μm. **(b)** The immunofluorescence of Ki67 and SOX9 in MDLCs-formed endometrial organoids after 3 cycles culture. **(c)** The immunofluorescence of ERα in MDLCs-derived organoids after 3 cycles culture.

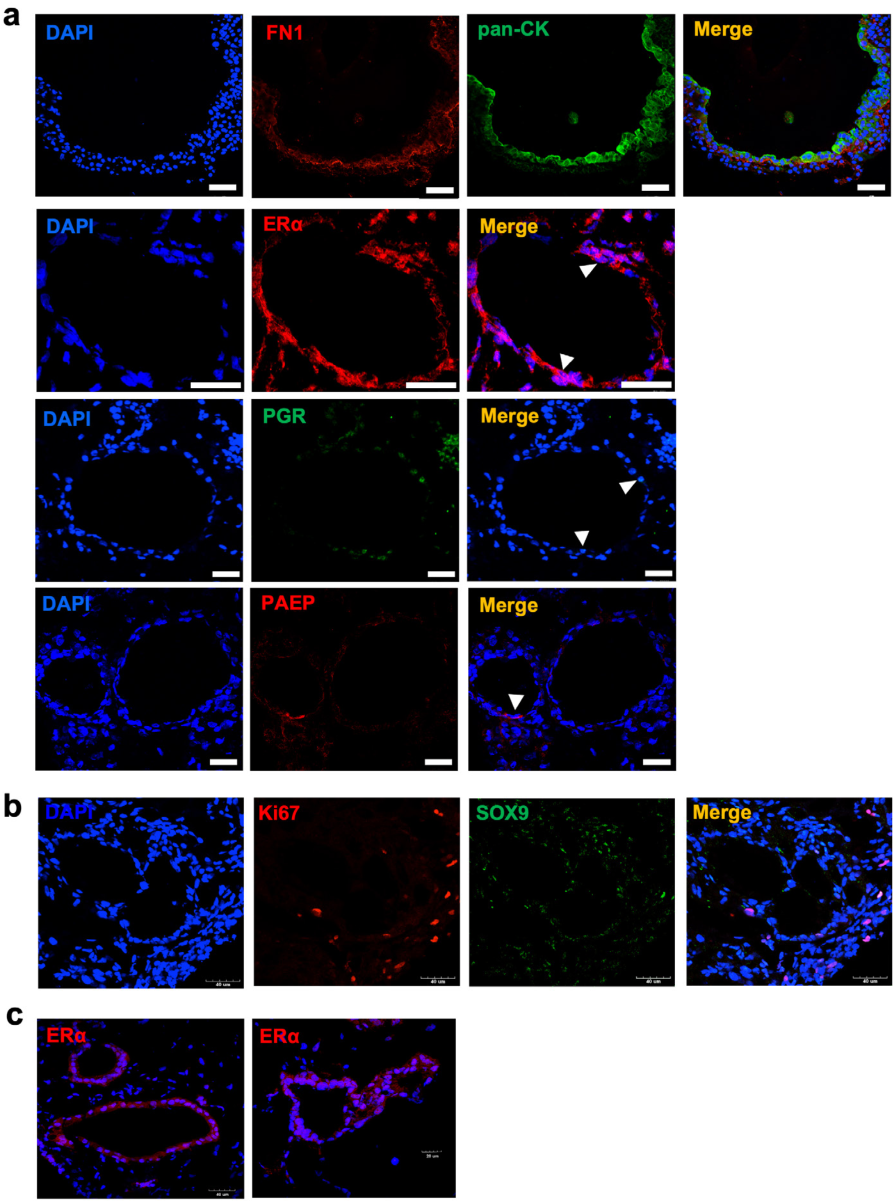

Supplementary Figure 5.

Cytotoxicity evaluation of GelMA hydrogel. **(a)** Representative live-dead staining fluorescent images of MDLCs embedded in the GelMA hydrogel at different time points (day 1, 3, 5). **(b)** Quantitative analysis of the cell viability in the GelMA hydrogel. The cells in the hydrogel showed a viability of 93.68 ± 2.14%, 93.84 ± 1.89% and 87.03 ± 1.73% at day 1, 3, 5, respectively. There was no cytotoxic effect of GelMA hydrogel on the trapped MDLCs. The results represent mean ± SEM for four independent experiments. **(c)** Cell viability of MDLCs cultured in ExM and extract of GelMA at different time points (day 1, 3, 5) by CCK-8 assay. GelMA hydrogel did not inhibited cell viability of MDLCs. The results represent mean ± SEM for three independent experiments. ns, $p > 0.05$.

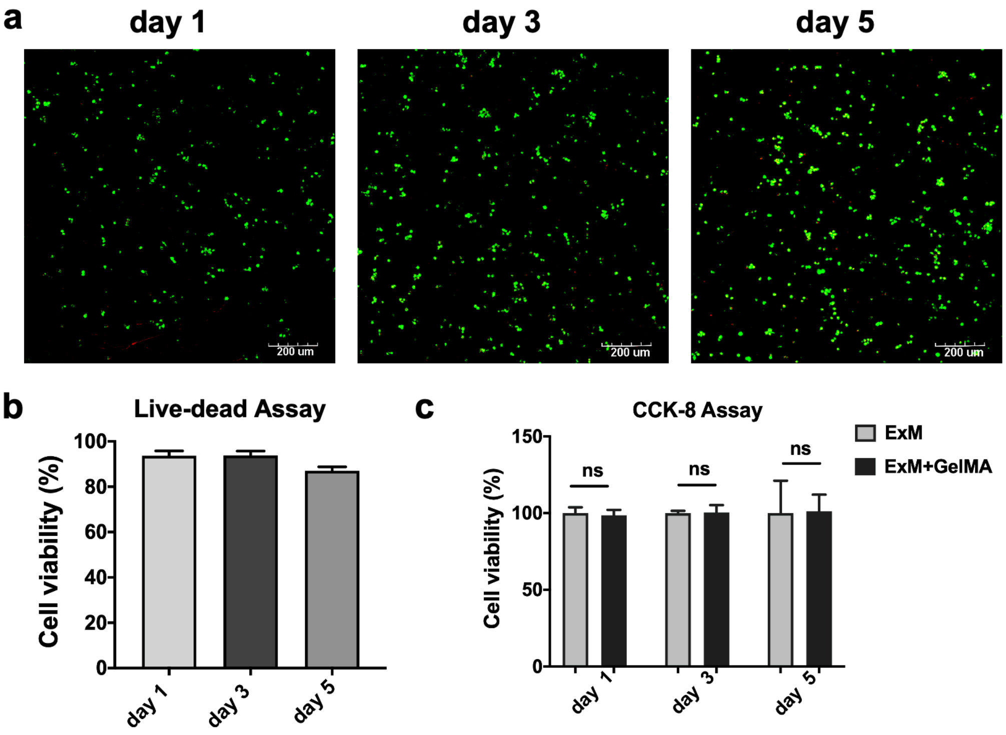

Supplementary Figure 6.

(a) The identification of H-LAMIN and pan-CK co-expressed cells (epithelium) in the endometrium of rats after repair for 6 weeks. **(b)** The expression levels of epithelial (*KRT8*) and stromal (*VIM*, *FN1*) makers in MDLCs-derived cells and NES. **(c)** The expression levels of specific markers of endometrium (*ERα*, *PGR* and *PAEP*) in MDLCs-derived cells and NES. **(d)** The expression levels of vascular markers (*VEGFA* and *vWF*) in MDLCs-derived cells and NES.

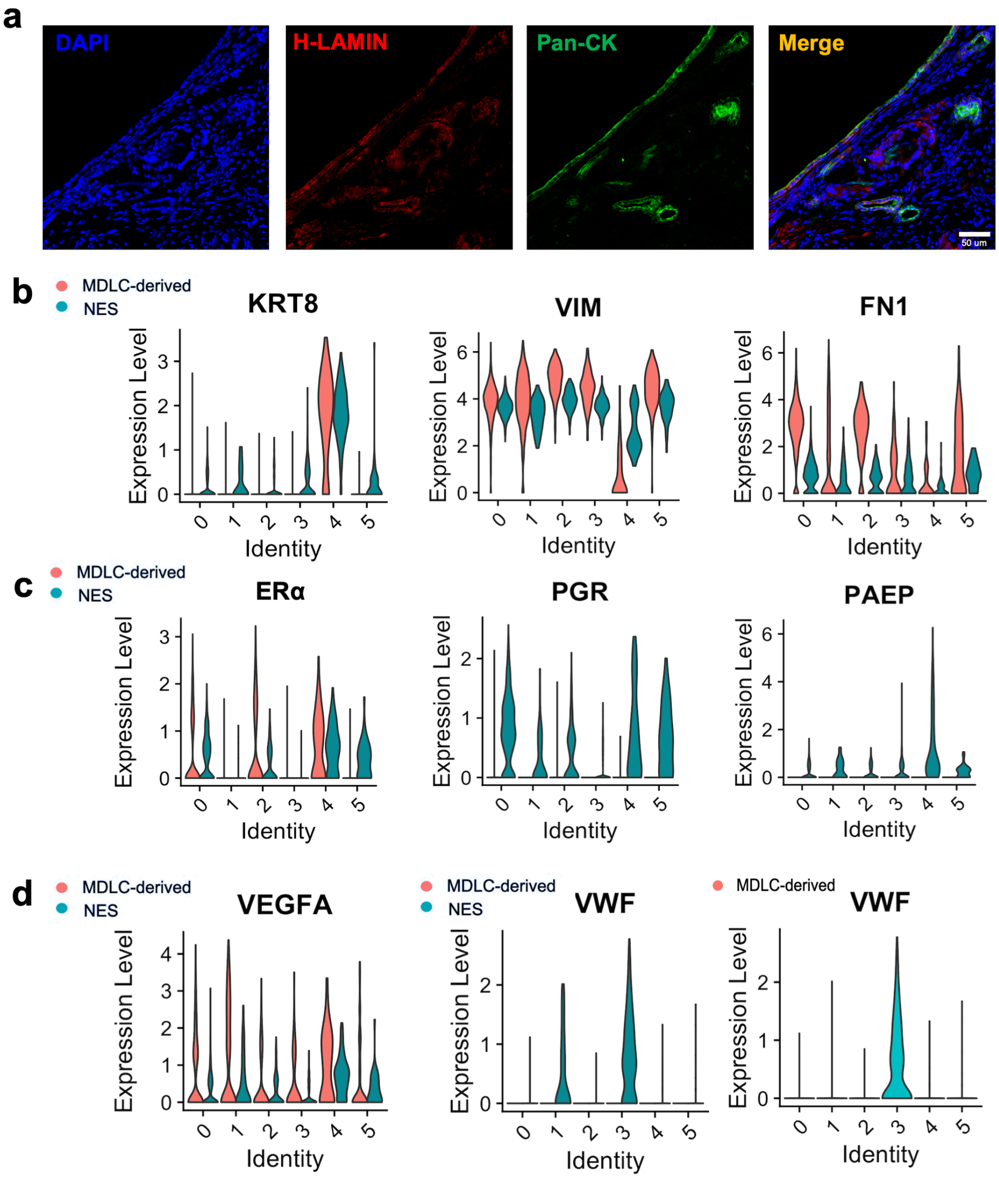

Supplementary Table 1.

Primers Sequences for qPCR

Primers	Forward	Reverse
GAPDH	TGACGCTGGGGCTGGCATTG	GGCTGGTGGTCCAGGGGTCT
OCT4	CAGTGCCCGAAACCCACAC	GGAGACCCAGCAGCCTCAAA
SOX2	ATGTCCCAGCACTACCAGAGC	GTGTGGATGGGATTGGTGTTCTC
WT1	GGGTACGAGAGCGATAACCA	TCTCACCAGTGTGCTTCCTG
OSR1	CCTTCCTTCAGGCAGTGAAC	CGGCACTTTGGAGAAAGAAG
BRACHYURY	GTGCTGTCCCAGGTGGCTTACAG ATG	CCTTAACAGCTCAACTCTAACTACTT G
MIXL1	ACGTCTTTCAGCGCCGAACAG	TTGGTTCGGGCAGGCAGTTCA
LHX1	ATCCTGGACCGCTTTCTCTT	GTACCGAAACACCGGAAGAA
PAX2	CAAAGTTCAGCAGCCTTTCC	CCACACCACTCTGGGAATCT
VIMENTIN	TCTGGATTCACTCCCTCTGGTT	ATCGTGATGCTGAGAAGTTTCG
KERATIN8	ATCAGCTCCTCGAGCTTCTC	TCCAGGAACCGTACCTTGTC
WNT7A	CTCCGGATCGGTGGCT	CCCATTTGTGAGCCTTCTCCT
SIX2	CTGGAGAGCCACCAGTTCTC	GCTGCGACTCTTTTCCTTGA
SOX9	GGCGGAGGAAGTCGGTGAAGAA	GCTCATGCCGGAGGAGGAGTGT
HOXA10	CTTCCGAGAGCAGCAAAGCCTC	TCCAGTGTCTGGTGCTTCGTGT
HOXA11	CAGCAGAGGAGAAAGAGCGGC	TCGGATCTGGTACTTGGTATAGG

Supplementary Table 2.

List of immunolabelling reagent used in this study

Reagent	Source	Identifier	Application	Dilution
**Primary Antibodies**				
pan-Cytokeratin	Abcam	Cat#ab7753	IF	1: 1000
KERATIN8	Abcam	Cat#ab53280	IF	1: 200
VIMENTIN	Dako	Cat#103465-002	IF	1: 50
PAX2	Abcam	Cat# ab79389	IF	1: 200
SOX9	Abcam	Cat# ab76997	IF	1: 100
Ki67	Abcam	Cat# ab16667	IF	1: 250
ERα	Abcam	Cat#ab32063	IF	1: 200
PGR	Abcam	Cat#ab32085	IF	1: 100
PAEP	Abcam	Cat#ab53289	IF	1: 100
Human LAMIN A+C	Abcam	Cat# ab108595	IF	1: 250
Fibronectin 1	Abcam	Cat# ab2413	IF	1: 200
KERATIN7	Abcam	Cat# ab181598	IHC	1: 8000
CD31	Abcam	Cat# ab182981	IHC	1: 2000
**Secondary Antibodies**				
Alexa Fluor 488 donkey anti mouse IgG (H+L)	Invitrogen	Cat# A21202		1: 5000
Alexa Fluor 546 goat anti rabbit IgG (H+L)	Invitrogen	Cat# A11035		1: 500

Goat anti- rabbit IgG H&L (HRP)	Jackson	Cat# 111035003	1: 500
**Nuclear and cell membrane staining**			
DAPI	Beyotime	Cat# C1002	1: 1000
DiI	Invitrogen	Cat# D282	1: 500

Code used for bulk RNA-seq analysis:

```
library(DESeq2)
library(pheatmap)
cts <- read.csv("hUiPSCs vs CHIR 36h vs bFGF+RA 20200601.csv",row.names = 1)
meta <- data.frame("Group" = factor(c(rep("hUiPSCs",3),rep("CHIR
36h",3),rep("bFGF+RA",3))),row.names = colnames(cts))
dds <- DESeqDataSetFromMatrix(countData = cts,colData = meta, design= ~Group)
dds$Group <- relevel(dds$Group,ref = "CHIR 36h")
vsd <- varianceStabilizingTransformation(dds, blind = FALSE)
p <- plotPCA(vsd,intgroup = c("Group"))
p +
  theme_classic() +
  geom_point(size=3)+
  theme(legend.position = "right",axis.title = element_text(size = 15)) +
  theme(legend.text=element_text(size=15),legend.title = element_text(size = 15))
ggsave("PCAplot.pdf")
dds <- DESeq(dds)
resultsNames(dds)
res <- results(dds,contrast = c("Group","bFGF+RA"," CHIR 36h"))
mcols(res,use.names=TRUE)
summary(res)
resOrdered<-res[order(res$pvalue),]
write.csv(as.data.frame(resOrdered),file = "bFGF+RA_vs_ CHIR 36h_res.csv")
res_p_0.05 <- as.data.frame(subset(resOrdered,(pvalue < 0.05)))
write.csv(res_p_0.05,file = "bFGF+RA_vs_ CHIR 36h_res_pval0.05.csv")
log2.norm.counts<- assay(vsd)[rownames(res_p_0.05),4:9]
annotation_col <- meta
pheatmap(log2.norm.counts,cluster_cols = F,cluster_rows = T,scale = "row",
         annotation_col = annotation_col,show_colnames = F,show_rownames = F,
         filename = "bFGF+RA_vs_CHIR 36h.heatmap(pval<0.05).tiff",
         border_color = NA,
         cellwidth = 20,
         main = "CHIR 36h vs bFGF+RA",treeheight_row = 10,treeheight_col = 0)
```

Code used for scRNA-seq analysis:

Cellranger(v3.1.0) pipeline was run at default parameters, followed by Seurat(v4.0.1) analysis pipeline.

```
{
  library(Seurat)
  library(ggplot2)
  library(dplyr)
  library(tibble)
```

```
  library(data.table)
}
# read in MDLC sample which is sequenced twice, combine them.

{
  human <- CreateSeuratObject(Read10X('~/lab/workplace/work21 sc gonglin/data/human_MDLC_20201125N/filtered_feature_bc_matrix/'),
                              "MDLC.human")
  humanB <- CreateSeuratObject(Read10X('~/lab/workplace/work21 sc gonglin/data/human_MDLC_20201128NB/filtered_feature_bc_matrix/'),
                               "MDLC.human.B")
}

all(rownames(human)==rownames(humanB))
cnt_A <- human@assays$RNA@counts
cnt_B <- humanB@assays$RNA@counts
overlap <- intersect(colnames(human),colnames(humanB))
cA <- cnt_A[,match(overlap,colnames(cnt_A))]
cB <- cnt_B[,match(overlap,colnames(cnt_B))]
all(colnames(cA)==colnames(cB))
cnt_all <- cA+cB
humanAll <- CreateSeuratObject(cnt_all,project = "Human")

####seurat#####
setwd("~/lab/workplace/work21 sc gonglin/analysis/humanAll")
setwd("QC/")
{
  humanAll[["percent.mt"]] <- PercentageFeatureSet(humanAll, pattern = "^MT-")
  humanAll[["log10GenesPerUMI"]] <- log10(humanAll$nFeature_RNA) / log10(humanAll$nCount_RNA)
  humanAll$orig.ident <- humanAll@active.ident

  metadata <- humanAll@meta.data
  metadata$cells <- rownames(metadata)
  metadata <- metadata %>%
    dplyr::rename(sample = orig.ident,
                  nUMI = nCount_RNA,
                  nGene = nFeature_RNA)

  library(scater)

  qc.lib2 <- isOutlier(metadata$nUMI, log=TRUE, type="both")
  attr(qc.lib2, "thresholds")
  # lower     higher
  # 1105.391  12211.317
  qc.nexprs2 <- isOutlier(metadata$nGene, log=TRUE, type="lower")
```

```
  attr(qc.nexprs2, "thresholds")
  # lower    higher
  # 463.6098        Inf
  qc.mito2 <- isOutlier(metadata$percent.mt, type="higher")
  attr(qc.mito2, "thresholds")
  # lower    higher
  # -Inf 1.01317
  # 1.0 is too-low, we dont use mito to remove
  discard2 <- qc.lib2 | qc.nexprs2
  # Summarize the number of cells removed for each reason.
  DataFrame(LibSize=sum(qc.lib2), NExprs=sum(qc.nexprs2),
                  Total=sum(discard2))
  # LibSize     NExprs      Total
  # <integer> <integer> <integer>
  #    1         111           0          111
  ##filtering##
  humanAll <- humanAll[,!discard2]
  dim(humanAll)
  humanAll <- NormalizeData(humanAll, normalization.method = "LogNormalize", sc
ale.factor = 10000)
  humanAll <- FindVariableFeatures(humanAll, selection.method = "vst", nfeatures
= 2000)
  # Identify the 10 most highly variable genes
  top10 <- head(VariableFeatures(humanAll), 10)
  # plot variable features with and without labels
  plot1 <- VariableFeaturePlot(humanAll)
  LabelPoints(plot = plot1, points = top10, repel = TRUE)
  ggsave("highvariableFeatures.pdf",width = 6)
  humanAll.genes <- rownames(humanAll)
  humanAll <- ScaleData(humanAll, features = humanAll.genes)
  humanAll <- RunPCA(humanAll, features = VariableFeatures(object = humanAll))
  VizDimLoadings(humanAll, dims = 1:2, reduction = "pca")
  ggsave("PCAcomponent.pdf",width = 6)
} #QC
DimPlot(humanAll, reduction = "pca")
ggsave("pca_dimplot.pdf")
ElbowPlot(humanAll,ndims = 50 )
ggsave("pca_elbowplot.pdf")

save(humanAll,file="~/lab/workplace/work21 sc gonglin/data/humanAll.Rdata")


####intergrate MDLC with Normal Endometrium in Secretory Stage#####

load("~/lab/workplace/work21 sc gonglin/data/humanAll.Rdata")
```

```
merged <-list(humanAll,NP)
#### with NS only ####
setwd("~/lab/workplace/work21 sc gonglin/analysis/humanAll/withNS")
load("~/lab/workplace/work21 sc gonglin/data/humanAll.Rdata")
meta <- read.table('~/lab/workplace/work21 sc gonglin/data/Normal_meta.txt',header
= T)
counts <- read.table( '~/lab/workplace/work4\ scRNA-seq/WBB_NS_201907.txt',row.n
ames = 1,header = T,sep = "\t")
NSmeta <- meta[grep("_NS",meta$Cell),]
NSmeta$Cell[!NSmeta$Cell %in%colnames(counts)]
NSmeta <- NSmeta[NSmeta$Cell %in%colnames(counts),]
counts <- counts[,NSmeta$Cell]
all(NSmeta$Cell == colnames(counts))
rownames(NSmeta) <- NSmeta$Cell
NS <- CreateSeuratObject(counts =counts,project = "NS",meta.data = NSmeta)
Idents(NS) <- NS@meta.data$Celltype
merged <-list(humanAll,NS)

##############

# normalize and identify variable features for each dataset independently
merged<- lapply(X = merged, FUN = function(x) {
  x <- NormalizeData(x)
  x <- FindVariableFeatures(x, selection.method = "vst", nfeatures = 2000)
})

# select features that are repeatedly variable across datasets for integration
features <- SelectIntegrationFeatures(object.list = merged)
anchors <- FindIntegrationAnchors(object.list = merged, anchor.features = features)
# this command creates an 'integrated' data assay
merged <- IntegrateData(anchorset = anchors)
# specify that we will perform downstream analysis on the corrected data note tha
t the original
# unmodified data still resides in the 'RNA' assay
DefaultAssay(merged ) <- "integrated"

# proprocess and PCA
merged <- merged %>%
  ScaleData(verbose = FALSE) %>%
  RunPCA(pc.genes = VariableFeatures(merged), verbose = FALSE)
ElbowPlot(merged,ndims = 30 )
merged <- merged %>%  RunUMAP(reduction = "pca", dims = 1:10)

merged$source <- c(rep("MDLC",ncol(humanAll)),rep("NS",ncol(NS)))

options(repr.plot.height = 5, repr.plot.width = 12)
```

```
p1 <- DimPlot(object = merged, reduction = "pca", pt.size = .1, group.by = "source
")
p2 <- VlnPlot(object = merged, features = "PC_1", group.by = "source",  pt.size =
0)
plot_grid(p1,p2)
ggsave("pca_intergreted.pdf",width = 10)

# rename few cluster in NS sample, group in Major Celltype
NScell <- colnames(NS)

cluster_name <- levels(Idents(merged))
names(cluster_name) <- levels(Idents(merged))

cluster_name[grep("Vascular_muscle",cluster_name)] <- "VascularMuscle"
cluster_name[grep("STROMA",cluster_name)] <- "Stroma"
cluster_name[grep("ENDO",cluster_name)] <- "Endothelium"
cluster_name[grep("EPI",cluster_name)] <- "Epithelium"

merged <- RenameIdents(merged, cluster_name)
merged$orig.ident <- merged@active.ident

### cluster the merged object ####
ElbowPlot(merged,ndims = 50 )
merged <- RunUMAP(merged, dims = 1:10)
merged <- RunTSNE(merged, dims = 1:10)
merged <- FindNeighbors(merged, dims = 1:10)
merged <- FindClusters(merged, resolution = 0.1)
cluster_name <- levels(Idents(merged))
names(cluster_name) <- levels(Idents(merged))
cluster_name["6"] <- "0"
merged <- RenameIdents(merged, cluster_name)
merged.markers <- FindAllMarkers(merged, only.pos = TRUE, min.pct = 0.25, logf
c.threshold = 0.25)
erged.markers.top50 <- merged.markers %>% group_by(cluster) %>% top_n(n = 5
0, wt =avg_log2FC)
merged@active.ident <- merged$seurat_clusters
DoHeatmap(merged,features = merged.markers.top50$gene)
ggsave("clus6.markers50.hm.png",units = "cm",dpi=600)
prec <- table(merged@meta.data$source,merged@active.ident)
prec <- melt(prec)
colnames(prec) <- c("group","cluster","proportion")
PlotPiefacet(prec,"reclus")
ggsave("reclus.prop.pdf")

DimPlot(merged, reduction = "umap", group.by = "source", pt.size = .1) + coord_fix
ed()
```

```
ggsave("source.umap.pdf")

save(merged,file="mergeNShumanall.Rdata")


### plot markers ###

DefaultAssay(merged) <- "RNA"
setwd("~/lab/workplace/work21 sc gonglin/analysis/humanAll/withNS/NS_humanall/RNA_fea")
#epi
library(RColorBrewer)
FeaturePlot(merged,c("KRT8","KRT18"))  &
   coord_fixed()
ggsave("Epi.featureplot.tiff",width = 7,height = 9)
VlnPlot(merged,c("KRT8","KRT18"),pt.size = 0)
ggsave("Epi.vlnplot.tiff",width = 7,height = 5)
VlnPlot(merged,c("KRT8","KRT18"),pt.size = 0,split.by = "source")
ggsave("Epi.compare.vlnplot.tiff",width = 7,height = 5)
#stroma
FeaturePlot(merged,c("FN1","VIM"))  &
  coord_fixed()
ggsave("Stroma.featureplot.tiff",width = 12,height = 10)
VlnPlot(merged,c("FN1","VIM"),pt.size = 0)
ggsave("Stroma.vlnplot.tiff",width = 7,height = 5)
VlnPlot(merged,c("FN1","VIM"),pt.size = 0,split.by = "source")
ggsave("Stroma.compare.vlnplot.tiff",width = 7,height = 5)
#vessel
FeaturePlot(merged,c("VEGFA","VWF"))  &
   coord_fixed()
ggsave("vacularendo.featureplot.tiff",width = 8,height = 9)
VlnPlot(merged,c("VEGFA","VWF"),pt.size = 0)
ggsave("vacularendo.vlnplot.tiff",width = 7,height = 5)
VlnPlot(merged,c("VEGFA","VWF"),pt.size = 0,split.by = "source")
ggsave("vacularendo.compare.vlnplot.tiff",width = 7,height = 5)
#endometrium

FeaturePlot(merged,c("ESR1","PGR","PAEP")) &
   coord_fixed()
ggsave("EM.featureplot.tiff",width = 8,height = 8)
VlnPlot(merged,c("ESR1","PGR","PAEP"),pt.size = 0)
ggsave("EM.vlnplot.tiff",width = 7,height = 5)
VlnPlot(merged,c("ESR1","PGR","PAEP"),pt.size = 0,split.by = "source")
ggsave("EM.compare.vlnplot.tiff",width = 7,height = 5)
```

```
m <- c("SOX9")
FeaturePlot(merged,m)     &
   coord_fixed()
ggsave("Stem.featureplot.tiff",width = 8,height = 8)
VlnPlot(merged,m,pt.size = 0)
ggsave("stem.vlnplot.tiff",width = 7,height = 5)
VlnPlot(merged,m,pt.size = 0,split.by = "source")
ggsave("stem.compare.vlnplot.tiff",width = 7,height = 5)
```

Copy
